# Intramolecular interactions stabilizing compact conformations of the intrinsically disordered kinase-inhibitor domain of Sic1: a molecular dynamics investigation

**DOI:** 10.3389/fphys.2012.00435

**Published:** 2012-11-22

**Authors:** Matteo Lambrughi, Elena Papaleo, Lorenzo Testa, Stefania Brocca, Luca De Gioia, Rita Grandori

**Affiliations:** Department of Biotechnology and Biosciences, University of Milano-BicoccaMilan, Italy

**Keywords:** intrinsically disordered proteins, Sic1, electrostatic interactions, cyclin-dependent kinase, molecular dynamics simulations, electrospray ionization mass spectrometry

## Abstract

Cyclin-dependent kinase inhibitors (CKIs) are key regulatory proteins of the eukaryotic cell cycle, which modulate cyclin-dependent kinase (Cdk) activity. CKIs perform their inhibitory effect by the formation of ternary complexes with a target kinase and its cognate cyclin. These regulators generally belong to the class of intrinsically disordered proteins (IDPs), which lack a well-defined and organized three-dimensional (3D) structure in their free state, undergoing folding upon binding to specific partners. Unbound IDPs are not merely random-coil structures, but can present intrinsically folded structural units (IFSUs) and collapsed conformations. These structural features can be relevant to protein function *in vivo*. The yeast CKI Sic1 is a 284-amino acid IDP that binds to Cdk1 in complex with the Clb5,6 cyclins, preventing phosphorylation of G1 substrates and, therefore, entrance to the S phase. Sic1 degradation, triggered by multiple phosphorylation events, promotes cell-cycle progression. Previous experimental studies pointed out a propensity of Sic1 and its isolated domains to populate both extended and compact conformations. The present contribution provides models for compact conformations of the Sic1 kinase-inhibitory domain (KID) by all-atom molecular dynamics (MD) simulations in explicit solvent and in the absence of interactors. The results are integrated by spectroscopic and spectrometric data. Helical IFSUs are identified, along with networks of intramolecular interactions. The results identify a group of putative hub residues and networks of electrostatic interactions, which are likely to be involved in the stabilization of the globular states.

## Introduction

Intrinsically disordered proteins (IDPs) are a class of promiscuous proteins that do not possess a well-defined three-dimensional (3D) structure in solution. Several IDPs or disordered domains can fold into ordered structures upon interactions with binding partners (Dyson and Wright, 2005; Espinoza-Fonseca, 2009b), although cases are known in which structural disorder is retained also in the bound state (Tompa and Fuxreiter, 2008; Meszaros et al., 2011). IDPs are very common in nature and notably in eukaryotes, where ~50% of the proteins are predicted to contain long disordered regions, and ~30% are classified as IDPs (Oldfield et al., 2005; Uversky and Dunker, 2010). These data are consistent with the observation that IDPs often play key regulatory roles in biological processes (Uversky et al., 2000; Uversky, 2002; Dyson and Wright, 2005; Tompa, 2005; Uversky and Dunker, 2010).

Most IDPs can transiently populate partially structured conformations in their unbound state and display intrinsically folded structural units (IFSUs). These elements are more often helical and are thought to provide seeds for binding interfaces (Sivakolundu et al., 2005; Espinoza-Fonseca et al., 2007, 2012; Belle et al., 2008; Espinoza-Fonseca, 2009a; Wright and Dyson, 2009; Kjaergaard et al., 2010; Norholm et al., 2011). Moreover, unbound IDPs can populate collapsed, globular conformations, stabilized by intramolecular interactions of both electrostatic and hydrophobic nature (Marsh et al., 2007; Espinoza-Fonseca, 2009a; Wostenberg et al., 2011).

Considerable efforts have been devoted to model IDP structural and dynamic properties at the atomic level (Dyson and Wright, 2005; Dunker et al., 2008; Turoverov et al., 2010; Fisher and Stultz, 2011). The understanding of IDP molecular recognition requires not only the description of the bound states (Morin et al., 2006; Receveur-Bréchot et al., 2006; Espinoza-Fonseca, 2009b; Hazy and Tompa, 2009; Wright and Dyson, 2009), but also the characterization of the elusive and heterogeneous unbound states (Dunker et al., 2008; Salmon et al., 2010; Fisher and Stultz, 2011; Szasz et al., 2011; Bernado and Svergun, 2012; Schneider et al., 2012). Atomistic or coarse-grained molecular dynamics (MD) simulations have proven suitable to describe the conformational landscape of IDPs or denatured proteins, identifying intrinsic or residual structure (Espinoza-Fonseca et al., 2007; Espinoza-Fonseca, 2009a, 2012; Yoon et al., 2009; Rauscher and Pomes, 2010; Cino et al., 2011; Fisher and Stultz, 2011; Qin et al., 2011; Ganguly et al., 2012; Lindorff-Larsen et al., 2012).

**Figure 1:**
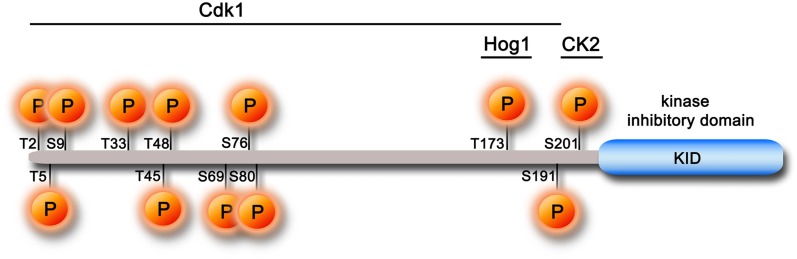
**Schematic representation of the full-length Sic1 protein and its principal phosphorylation sites**. The minimal functional KID fragment, corresponding to the last 70 residues of the protein, is indicated as a blue box. The main kinases that regulate Sic1 activity in the cell cycle are indicated, along with their phosphorylation sites on the whole protein.

Several IDPs are involved in cell-cycle regulation (Galea et al., 2008). Among these, cyclin-dependent kinase inhibitors (CKIs) modulate the activity of cyclin-dependent kinases (Cdks) playing a key role in the regulation of the eukaryotic cell cycle (Besson et al., 2008; Barberis, 2012). The IDP Sic1 is a yeast CKI that regulates the timing of entrance into S-phase by inhibition of the Clb5-6/Cdk1 complex (Schwob et al., 1994). The G1-to-S transition is executed only upon ubiquitin-dependent Sic1 degradation by the proteasome. Sic1 is targeted for destruction by the Skp1-Cul1-F-box complex, SCF^cdc4^. The interaction with SCF is triggered by Sic1 multiple phosphorylation in its N-terminal region (Nash et al., 2001; Mittag et al., 2008; Koivomagi et al., 2011) and inhibited by the stress-response phosphorylation on T173 (Escote et al., 2004; Yaakov et al., 2009). Such a network of regulatory functions contributes to effective cell division and genome integrity (Verma et al., 1997; Mendenhall and Hodge, 1998; Deshaies and Ferrell, 2001; Nash et al., 2001; Barberis, 2012; Barberis et al., 2012) (Figure 1). Sic1 is also involved in other regulatory processes, such as exit from mitosis (Lopez-Aviles et al., 2009) and coupling of cell growth to cell-cycle progression (Coccetti et al., 2004; Barberis et al., 2007).

Sic1 is 284-residue long and, although disordered in its whole length, it can populate conformations with various degrees of compactness, revealing global structural properties more typical of a collapsed chain than of a random coil (Brocca et al., 2009, 2011b). Its free state is characterized by tertiary and secondary structures (Brocca et al., 2009). The little content in secondary structures (mainly helical) is distributed quite uniformly throughout the polypeptide chain, although the C-terminus is slightly more ordered than the N-terminus (Brocca et al., 2011a,b). Conformational analysis by electrospray-ionization mass spectrometry (ESI-MS) and limited proteolysis suggests that the C-terminal moiety also contains more tertiary structure than its complementary N-terminal region (Brocca et al., 2011b; Testa et al., 2011b). The last 70 residues have been identified as the minimal protein fragment for *in vivo* Cdk1 inhibition (Verma et al., 1997; Hodge and Mendenhall, 1999; Nash et al., 2001) and have been proposed to be structurally and functionally related to the kinase inhibitory domain (KID) of the mammalian tumor-suppressor p21 and p27 Kip/Cip proteins (Toyoshima and Hunter, 1994; Barberis et al., 2005) (Figure 1). An X-ray structure of the p27 ternary complex with Cdk2-cyclin A is available (Russo et al., 1996). No structural data are available for the yeast ternary complex, but a model was developed based on the template of the mammalian complex (Barberis et al., 2005).

This work investigates the conformational ensemble of Sic1 KID by integrating all-atom explicit solvent MD simulations with experimental data to achieve a model of the compact conformations populated by the protein in the absence of interactors. The results identify regions that are likely to be characterized by intrinsic secondary and tertiary structure and point out a predominant role of electrostatic interactions in promoting protein compaction.

## Materials and methods

### Starting structures for MD simulations

An extended conformation of the Sic1 KID fragment (residues 215–284) was modeled by the *generated_extended.inp* module of the *Crystallography* & *NMR System* (CNS) software (Brunger, 2007) to avoid tertiary contacts and to allow the protein to rearrange during MD simulations. Starting from this structure, 10 different models were generated by the program *MODELLER*, including constraints derived from the prediction of secondary structure (Barberis et al., 2005). These models feature a main-chain root mean square deviation (RMSD) between 0.7 and 10 nm in pairwise comparisons. Both approaches were previously applied to other IDPs (Gardebien et al., 2006; Cino et al., 2011). In particular, the regions identified by at least two predictors (Barberis et al., 2005) were constrained to α-helices (T226-L238 and I244-I248). In fact, *MODELLER* can be used to generate structural models, satisfying spatial restraints. It employs knowledge-based probability density functions (PDFs) derived by statistical mechanics (Eswar et al., 2008). Among the models featuring no side-chain interactions, four models were selected as the starting structures of MD simulations based on the DOPE and GA341 *MODELLER* scores.

### Molecular dynamics simulations

MD simulations were performed using the 4.5.3 version of the *GROMACS* software (www.gromacs.org), implemented on a parallel architecture, using the GROMOS96 force field. The Sic1 KID models described above were used as starting structures for all-atom, explicit solvent, MD simulations, employing periodic boundary conditions. The Sic1 KID molecule was soaked in a dodecahedral box of Simple Point Charge (SPC) water molecules (Fuhrmans et al., 2010) with all the protein atoms at a distance equal or greater than 1.5 nm from the box edges.

Productive MD simulations were performed in the isothermal-isobaric (NPT) ensemble at 300 K and 1 bar, using an external bath with thermal and pressure coupling of 0.1 and 1 ps, respectively. The LINCS algorithm (Hess et al., 1997) was used to constrain heavy-atom bonds, allowing for a 2 fs time-step. Electrostatic interactions were modeled by the Particle-mesh Ewald summation scheme (Darden et al., 1993). Van der Waals and Coulomb interactions were truncated at 1.2 nm, a cutoff value previously used for IDP simulations and experimentally validated by comparison with electronic paramagnetic resonance and fluorescence data (Espinoza-Fonseca et al., 2008; Espinoza-Fonseca, 2009a). The non-bonded pair list was updated every 10 steps and conformations were stored every 2 ps. The simulations were carried out in the presence of Na^+^ and Cl^−^ counterions, to simulate a physiological ionic strength (150 mM), according to a protocol previously employed for other IDPs (Espinoza-Fonseca, 2009a; Arrigoni et al., 2012). The length of each simulation (replicate, r.) ranged from 50 (r.1–r.6 and r.9) to 100 ns (r.7 and r.8). The first 0.5 ns of each replicate were discarded to avoid artifacts arising from the preparation procedure. A concatenated macro-trajectory, including 9 replicates, for a total duration of 545.5 ns, was generated to obtain a conformational ensemble of Sic1 KID in solution.

### Analysis of MD simulations

The secondary structure content of the models was calculated by the *DSSP* program (Kabsch and Sander, 1983), along with a residue-dependent profile of secondary structure persistence. Salt-bridge, aromatic, and hydrophobic interactions were analyzed as previously described (Tiberti and Papaleo, 2011; Arrigoni et al., 2012). In particular, a persistence cutoff of 20% and a distance cutoff of 0.5 nm were employed. Aromatic interactions were analyzed using a 0.6 nm distance cutoff.

### Analysis of salt-bridge interaction networks

Salt-bridge interactions were also analyzed by the *Pymol* plugin *xPyder*, representing pairwise relationships extracted from protein structures by two-dimensional matrices (Pasi et al., 2012). In particular, the module for network analysis implemented in *xPyder* has been employed. A network is described as a set of points (nodes) and connections between them (edges), according to Vishveshwara et al. (2009). A path is defined as a sequence of nodes for which an edge always exists between two consecutive nodes of the path. A matrix describing the persistence of each salt bridge in the MD ensemble (i.e., the number of trajectory frames in which the interaction was present divided by the total number of frames) was used as input file. The program represents each residue of the matrix as a node of a simple, weighted graph connected by edges, whose weights are defined by the persistence of the interaction in the MD ensemble.

Residues connected by more than 3 edges to their neighbors (Brinda and Vishveshwara, 2005) are referred to as hubs of the interaction network. Hubs in protein networks are known to play key roles in protein structure and function (Vishveshwara et al., 2009; Angelova et al., 2011). The connected components of the graph were also calculated. These are isolated sub-graphs in which all the edges are linked by at least one path, but no path exists between the nodes of the connected component and the rest of the graph. This analysis allows us to identify different clusters of salt-bridge networks. Finally, all the possible paths existing between two nodes of the graph were calculated employing a variant of the depth-first search algorithm (Cormen et al., 2009), as implemented in *xPyder*. The searching procedure was carried out so that the same node is not visited more than once to avoid entrapment in cycles.

### Principal component analysis (PCA) and free energy landscape (FEL)

PCA highlights high-amplitude, concerted motions in MD trajectories, through the eigenvectors of the mass-weighted covariance matrix (C) of the atomic positional fluctuations (Amadei et al., 1993). Both all-atom and Cα-only matrices were calculated for the macro-trajectory. Given a reaction coordinate *q*_α_, the probability of finding the system in a particular state *q*_α_ is proportional to (*e*−^*G*(*q*α)/*kT*^), where *G*(*q*_α_) is the Gibbs free energy of that state. The FEL can be estimated from the equation *G*(*q*_α_) = −*kTln*[*P*(*q*_α_)], where *k* is the Boltzmann constant, *T* is the temperature of the simulation and *P*(*q*_α_) is an estimation of the probability density function obtained from a histogram of the MD data. Considering two different reaction coordinates, for example *q* and *p*, the two-dimensional FEL can be obtained from the joint-probability distribution *P(q,p)* of the considered variables. In particular, the reaction coordinates considered in this study were the first and the second, as well as the first and the third cartesian principal components (PCs or eigenvectors) derived by the PCA procedure described above.

The root mean square inner product (RMSIP) is a measure of similarity between subspaces sampled by different trajectories and it was calculated for each pair of independent replicates, comparing the subspace described by the first 10 eigenvectors from PCA (Amadei et al., 1999) according to the formula

$$\text{RMSIP}=\frac{1}{D}\sum_{i=1}^{D}\sum_{j=1}^{D}\left(\eta_{i}^{A}\eta_{j}^{B}\right)$$

where η^*A*^_*i*_ and η^*B*^_*j*_ are the eigenvectors to be compared and D the number of eigenvectors considered.

### Cluster analysis

The structures belonging to each FEL basin were isolated and the pairwise main-chain RMSD matrix was calculated for each basin. The Gromos algorithm (Keller et al., 2010) was employed for clustering, using a cutoff of 0.4 nm. For each cluster, the structure with the lowest RMSD with respect to the other members of the cluster was selected as the average structure.

### Order parameter *O*

The order parameter *O* was calculated according to the formula (Fisher and Stultz, 2011):

$$O=\sum_{i\;=\;1}^{n}w_{i}\log_{2}\left[1+\sum_{j\;=\;1}^{n}w_{j}\exp\left(-\frac{D^{2}\left(s_{i},\;s_{j}\right)}{2\langle D^{2}\rangle }\right)\right]$$

where *D*^2^(*s*_*i*_, *s*_*j*_) is the Cα mean-square distance (MSD) between the structures *s*_*i*_ and *s*_*j*_, and 〈*D*^2^〉 is the average pairwise MSD due to the fluctuations of a typical protein structure at a specific temperature [according to Fisher and Stultz (2011), this value is 0.27 nm]. Protein conformations were extracted from the MD macro-trajectory after clustering, by the algorithm using three different values of RMSD cutoff (0.3, 0.4, and 0.5 nm). The average structure of each cluster was extracted to create an ensemble of protein conformations and to calculate the order parameter *O*. The weights associated with each conformation were set as the relative size of each cluster, dividing the number of structures assigned to a cluster by the total number of frames in the trajectory. The results obtained by using the different cutoffs are reported in Table 2.

### Mass spectrometry

ESI-MS experiments were performed on a hybrid quadrupole-time-of-flight mass spectrometer (QSTAR ELITE, Applied Biosystems, Foster City, CA) equipped with a nano-ESI sample source. Samples of 10 μM recombinant Sic1 KID fused to a C–terminal His_6_ tag (Brocca et al., 2011b) in 50 mM ammonium acetate, pH 6.5 were loaded in metal-coated borosilicate capillaries for nanospray (Proxeon, Odense, DK) with medium-length emitter tip of 1 μm internal diameter. The instrument was calibrated using the renine inhibitor (1757.9 Da) (Applied Biosystems) and its fragment (109.07 Da) as standards. Spectra were acquired in the 500–2000 *m/z* range, with accumulation time 1 s, ion-spray voltage 1200 V, declustering potential 80 V, keeping the instrument interface at room temperature. Spectra were averaged over a time period of 2 min. Data analysis was performed by the program *Analyst QS 2.0* (Applied Biosystems). Gaussian fitting of ESI-MS spectra was carried out on row data reporting ion relative intensity *versus* charge (Dobo and Kaltashov, 2001). These data were fitted by the minimal number of Gaussian functions leading to a stable fit. Fitting analyses were performed by the software *OriginPro 7.5* (Originlab, Northampton, MA).

## Results

**Figure 2:**
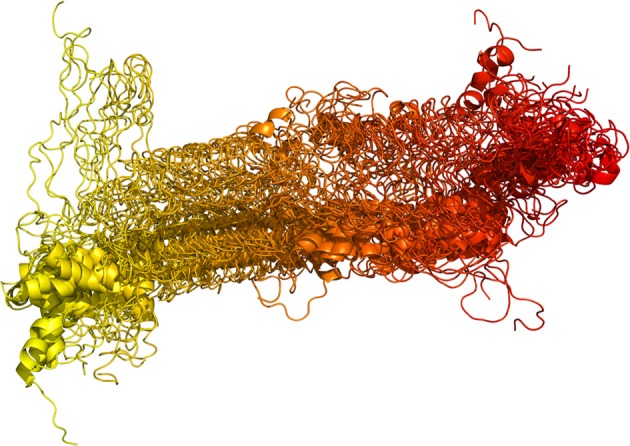
**Structural displacement of the Sic1 KID domain in the MD macro-trajectory**. The equilibrated parts of each replicate were concatenated in a single macro-trajectory of more than 0.5 μs, shown here. Snapshots are collected every 2 ns, overlaid and represented by a color gradient, from yellow (the starting structure of replicate 1) to red (the last frame of replicate 9).

Structural models of Sic1 KID (residues 215–284) in an extended conformation were generated as described in the “Materials and Methods”, satisfying constraints on secondary structure according to the previously published predictions (Barberis et al., 2005). This prediction is also in agreement with the average content of secondary structure indicated by circular dichroism (CD) (Brocca et al., 2011a) and Fourier-transform infrared (FT-IR) spectroscopy (Brocca et al., 2011b). Multiple, 50–100 ns, independent MD simulations were carried out starting from four extended models, collecting overall more than 500 ns of MD trajectories (Figure 2). The results of MD simulations were analyzed with reference to secondary structure content, solvent-accessible surface (SAS), and intramolecular interactions, as described in details in the following. Moreover, distinct conformational substates were identified in the simulated ensemble by integrating structural clustering, PCA and FEL calculations (Papaleo et al., 2009; Zhuravlev et al., 2009).

### Order parameter *O*

The order parameter *O* was calculated to evaluate the heterogeneity of the conformational ensemble described by the MD simulations (Fisher and Stultz, 2011). Indeed, this parameter can be considered as a quantitative measure of the disorder in a given structural ensemble. The *O* parameter was calculated on the average structures derived from cluster analysis on the MD ensemble. The results are reported in Table 1. The Sic1 KID fragment displays very low values of the *O* parameter, ranging from 0.141 to 0.156, depending on the number of clusters considered. The limit value of 0 applies to the ideal case of an infinite number of equally populated, different conformations. Therefore, these results indicate that Sic1 KID exists as a highly heterogeneous conformational ensemble, confirming the strong propensity of this fragment for structural disorder.

**Table 1:** **Order parameter estimated for the MD ensemble of Sic1 KID using three different cutoff values**.

**Cutoff of clustering**	**Number of clusters**	**Order parameter**
6	55	0.141
5	79	0.144
4	158	0.156

### Dynamic behavior

**Figure 3:**
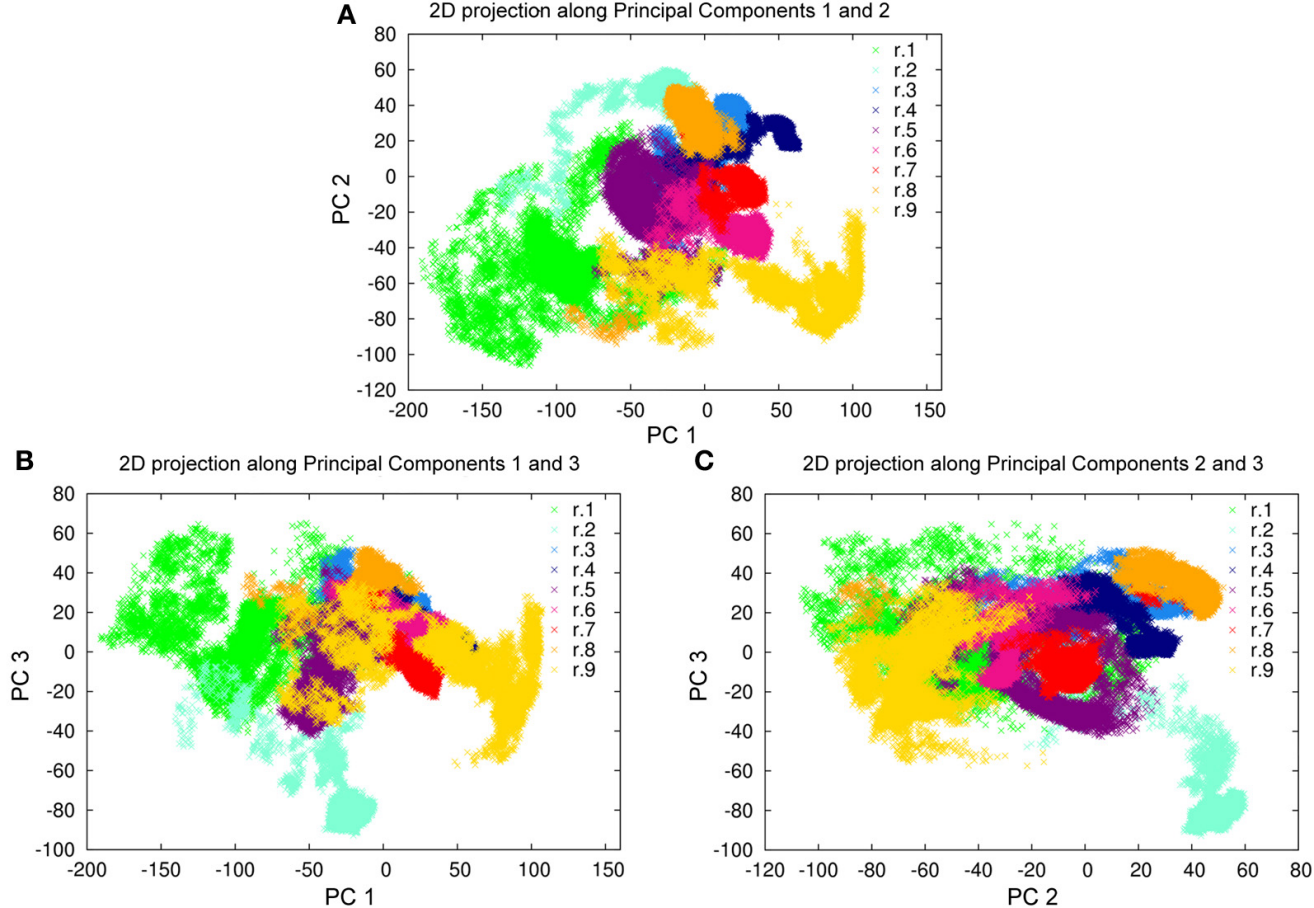
**Projections of the MD trajectories of Sic1 KID on the first 3 PCs derived from PCA of the macro-trajectory. (A)** First (*x* axis) and second (*y* axis) eigenvectors, **(B)** first (*x* axis) and third (*y* axis) eigenvectors, **(C)** second (*x* axis) and third (*y* axis) eigenvectors. The distinct contributions of the MD replicates are shown by different colors.

To evaluate the conformational sampling achieved by our MD investigation, and to better define the dynamic behavior of Sic1 KID, all-atom and Cα-only PCA were carried out on the macro-trajectory. PCA can provide an estimate of the conformational sampling achieved in a MD ensemble (Hess, 2002) and can describe the sampled conformational landscape, if combined to FEL calculations. The projections of the MD macro-trajectory on the first two PCs show an efficient sampling of the conformational space. In fact, distinct simulations sample different regions of the subspace described by the first two PCs, featuring partial overlap (Figure 3A). In particular, simulations starting from different initial models often populate the same basins, confirming that the MD ensemble provides a good description of Sic1 KID conformational landscape (Amadei et al., 1999; Hess, 2002; Papaleo et al., 2009). Similar results were also achieved analyzing the two-dimensional projections along the first and the third PCs, as well as along the second and the third ones (Figures 3B,C).

**Figure 4:**
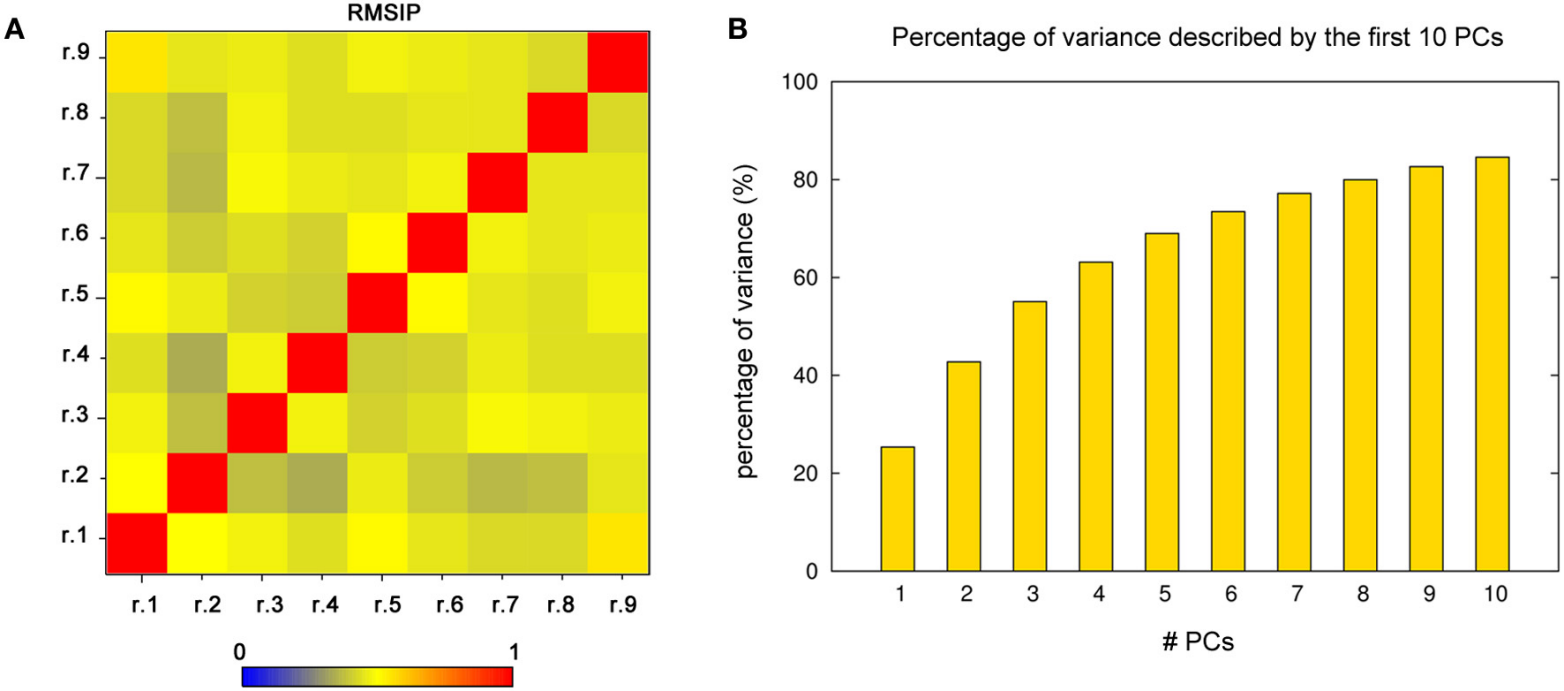
**PCA analysis. (A)** RMSIP matrix calculated for each pairwise comparison of the single replicates, using the conformational space described by the first 10 PCs. The RMSIP values are highlighted by a color gradient, from blue to red. **(B)** Cumulative percentage of the variance described by the first ten PCs. The first three (or five) PCs account together for more than 50% (or 70%) of the global motions of the domain.

To better quantify the trajectory overlap, the RMSIP of the first ten PCs between the different replicates (Figure 4A) was calculated as described in the “Materials and Methods”. In fact, RMSIP is an index of similarity between essential subspaces. In our simulations, the independent replicates with highest overlap have a RMSIP value higher than 0.5, and the average RMSIP is 0.44. Overall, the RMSIP analysis indicates satisfactory convergence, with a high overlap of the dynamics information provided by the simulations.

Since the first three PCs account alone for more than 50% of the global motion of Sic1 KID (Figure 4B), they can be used as the so-called *essential subspace* to describe the main dynamical properties of this domain. The projections on the first three PCs show that the protein has a very heterogeneous structural ensemble composed by several, different, highly populated conformations (Figure 3).

**Figure 5:**
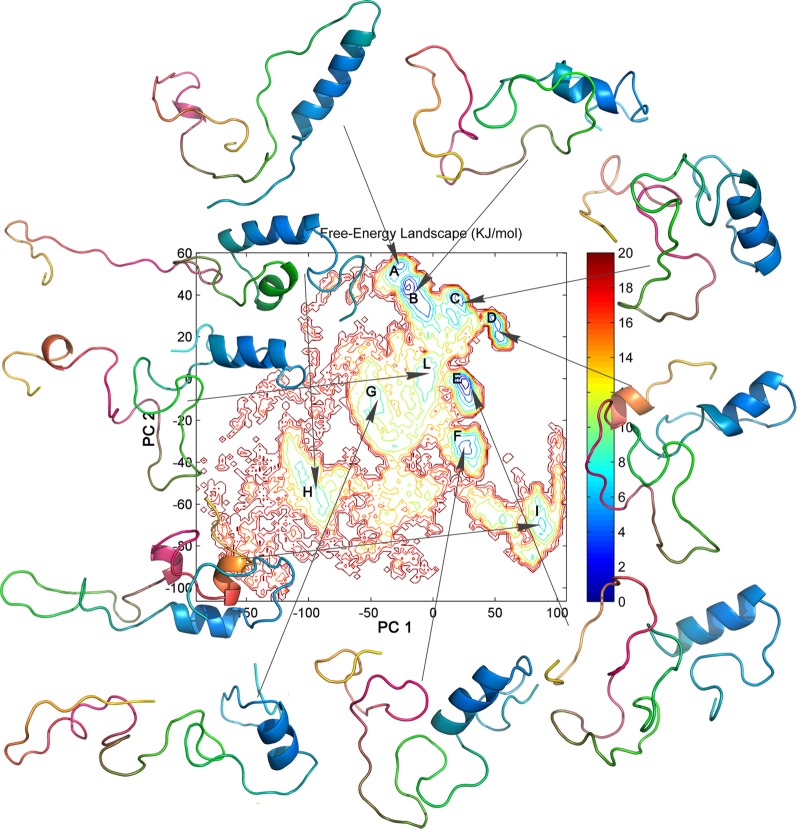
**FEL representation calculated using the first two PCs as reaction coordinates**. The major basins are labeled by capital letters (A to L). The free energy is given in kJ/mol and indicated by the color code shown on the figure. The average 3D structure identified for each conformational basin by structural clustering is represented as cartoon. The 3D structures are highlighted by a color gradient, from N-terminus (cyan) to C-terminus (yellow).

**Figure 6:**
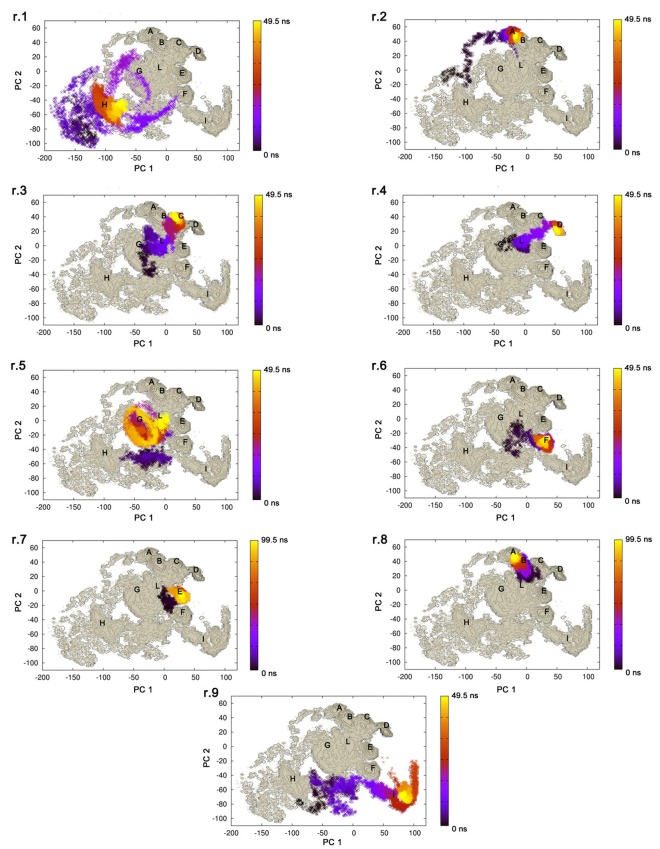
**Projection of the single replicate on the FEL**. The trajectories explored by each replicate are depicted on the FEL representation obtained using the first two PCs as reaction coordinates. The temporal evolution of each replicate is indicated by a color gradient, from black (0 ns) to yellow (the last frame of each replicate).

To characterize and isolate the different conformational states of the MD ensemble, the FEL was calculated (Zhuravlev et al., 2009) using the first two PCs as reaction coordinates (Figure 5). The average structure representative of each FEL basin is also reported in Figure 5. Other FEL representations were also calculated for comparison, using the first and the third, or the second and the third PCs (*data not shown*). It should be pointed out that these low-dimensional FEL representations applied to classical MD simulations are not sufficiently accurate to calculate the free-energy barriers and to describe the location of metastable states and barriers in details (Altis et al., 2008). Nevertheless, they can be very useful to describe the conformational landscape accessible to the molecule in the MD ensemble. In the FEL representation depicted in Figure 5, seven major (A, B, C, D, E, F, I) and three minor (G, H, L) conformational basins can be identified (Figure 5). The projections of the single trajectories on the FEL (Figure 6) show that each replicate can sample different conformational basins, in line with the data reported in Figure 3.

For the structures of each FEL basin, we have calculated the secondary structure content, the SAS values, as well as the networks of salt-bridge, aromatic, amino-aromatic, and hydrophobic interactions and their persistence (Tables 2, 3). The 3D average structures extracted from the ensemble trajectories have different structural properties, with less (A, G, H, L) and more compact (C, D, F, E) conformations, highlighting the intrinsic structural flexibility of Sic1 KID. Indeed, starting from completely extended models, the protein populates quite different conformational states, from extended random-coil-like conformations to highly compact structures.

**Table 2:** **Secondary structure content and SAS values**.

**Basin name**	**Helical content average (α–3_10_–π)**	**Helical content maximum (α–3_10_–π)**	**Total helical content average (%)**	**SAS average (nm^2^)**	**SAS minimum (nm^2^)**	**SAS maximum (nm^2^)**
A	16.42–0.01–1.23	23–6–7	27.15	60.10	53.45	67.63
B	7.35–1.89–0.94	22–7–10	15.71	56.32	49.70	70.87
C	10.65–0.09–0.08	16–6–6	17.14	53.57	47.84	68.94
D	13.2–0.01–0.12	16–5–4	20.00	54.71	50.05	60.05
E	10.54–0.16–0.11	16–5–6	15.71	55.94	50.05	68.32
F	7.33–1.97–0.06	21–11–6	14.28	55.31	48.70	68.81
G	10.89–0.02–0.12	22–8–6	17.14	58.72	53.10	75.83
H	17.75–0.01–0.03	21–5–6	25.71	65.24	60.25	71.26
I	14.51–0–0.23	24–0–6	22.85	57.58	53.08	64.29
L	12.11–0.07–0.26	15–9–6	18.57	60.78	53.87	71.74

The secondary structure content for each basin has been calculated considering all the different types of helical structures. Values are shown as number of residues or as percentage of the total number of residues in the Sic1 KID fragment. The minimum, maximum, and average SAS values are also displayed.

**Table 3:** **Hub residues identified for each conformational basin**.

**Basin**	**Hub residues**
A	K260, E266
B	D243, E245, D246, K254, E256, E259, R261, R262, E266, E267, K268, R270
C	E 223, E240, D265, R270
D	R233, E245, R261, D265, E267, R270
E	R270, D281
F	K268
G	K234, K254, R261, R270, E283
H	R262, E266
I	R262, D265, K268, R269, D281
L	E240, R261, D265, R270

The residues involved in three or more connections in the salt-bridge networks have been considered as hubs.

The here described principal motions of Sic1 KID identify distinct N- and C-terminal sub-domains. The first PC mainly accounts for the dynamics of the C-terminal region (yellow and hot-pink in the snapshots shown in Figure 5), whereas the second PC mostly describes the motions of the N-terminal region (cyan in the snapshots shown in Figure 5). These two motions dictate the major features of the dynamic behavior of Sic1 KID in our MD ensemble. In fact, these motions are related to the pairing of the C-terminal and N-terminal regions and are likely to reflect the transitions between an *open* and a *closed* state of the domain in the MD framework described here. The closed state is characterized by a long unstructured loop in the central region of the domain, approximately spanning residues 243–270 (green in the snapshots shown in Figure 5).

### Secondary structure content

The MD data were analyzed to identify putative IFSUs, i.e., regions that are characterized by at least transient secondary structure during the simulation time. The secondary structure content was calculated for each FEL basin and compared with experimental data obtained by FT-IR spectroscopy (Brocca et al., 2011b). The FT-IR spectra of Sic1 KID point out a high contribution of random-coil conformation (~40%) in addition to a ~30% of dynamic helical structures (α, 3_10_, and π helix). The average secondary structure content calculated for each MD basin is reported in Table 2. The data extracted from MD simulations are in overall good agreement with FT-IR data, although slightly under-estimated. Such a discrepancy is likely due to inherent limits of the *GROMOS96* force field when sampling α-helical conformations (Matthes and De Groot, 2009).

The structures belonging to basins A and H (Table 2) provide the best agreement with the available experimental data (Brocca et al., 2011b). The average structure derived from basin A displays a finger-like structure, composed by a long α-helix from residue L224 to residue R239. Instead, the average structure from basin H displays two α-helices, from Q227 to L238 and from I244 to T249.

### Intramolecular interactions

**Figure 7:**
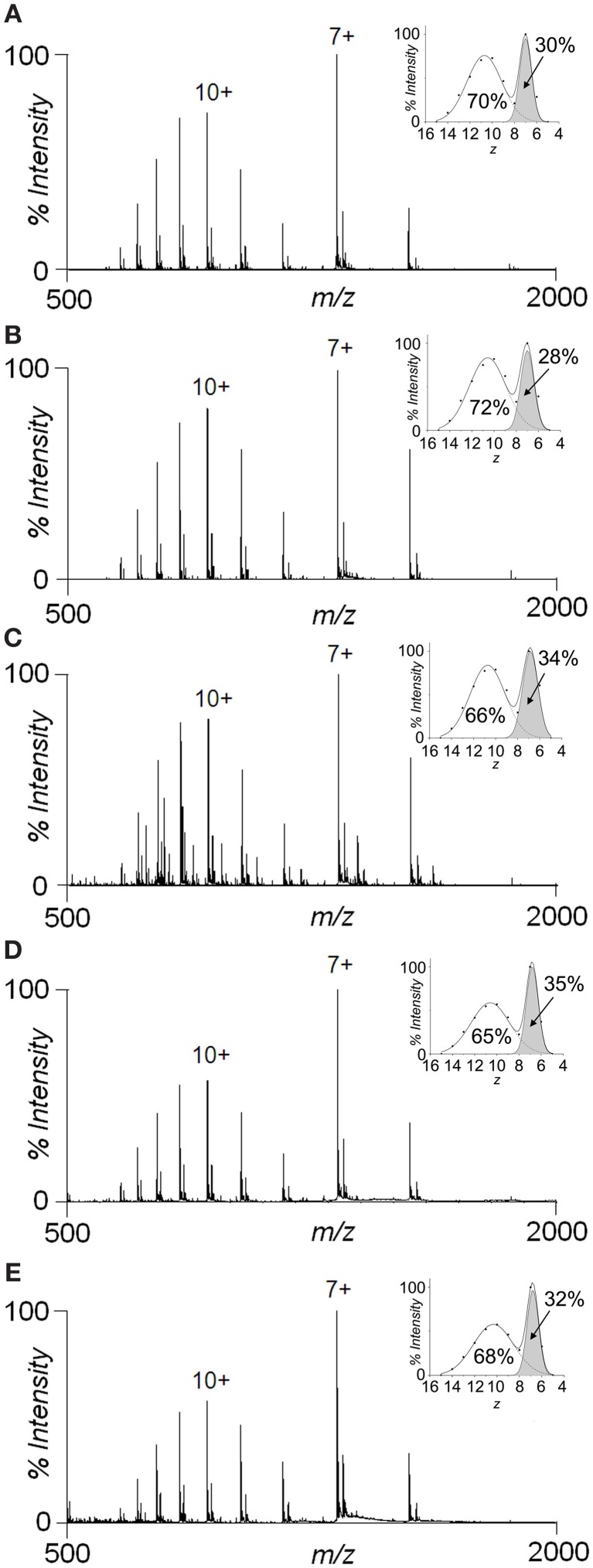
**Species distributions by ESI-MS and effects of organic solvents**. Nano-ESI-MS spectra of 10 μM protein in **(A)** 50 mM ammonium acetate, pH 6.5; **(B)** 50 mM ammonium acetate, pH 6.5, 30% acetonitrile; **(C)** 50 mM ammonium acetate, pH 6.5, 50% acetonitrile; **(D)** 50 mM ammonium acetate, pH 6.5, 30% methanol; and **(E)** 50 mM ammonium acetate, pH 6.5, 50% methanol. The main charge state of each component is labeled by the corresponding charge state (7+ for the compact form and 10+ for the extended form). The insets show the gaussian fitting of the CSDs upon transformation to an *x = z* abscissa axis.

To characterize the tertiary structure properties of the distinct conformations populated by Sic1 KID, the SAS values were calculated for the different structures of each FEL basins (Table 2). The average SAS values range from 53 to 65 nm^2^ (Table 2). These results can be compared with estimates obtained by ESI-MS, since the extent of ionization correlates with the SAS of the protein at the moment of its transfer to the gas phase (Testa et al., 2011a). The Sic1 KID fragment gives rise to bimodal charge-state distributions (CSDs) by non-denaturing ESI-MS (Brocca et al., 2011b) (Figure 7A), indicating coexistence of compact and extended conformations (Kaltashov and Abzalimov, 2008). The predominant component represents a highly extended state, while the minor component (apparent relative amount ~30%) represents a compact state that disappears upon acidification, as previously reported (Brocca et al., 2011b). The average SAS values derived from the ESI-MS data (Brocca et al., 2011b) are 59.78 nm^2^ for the compact state and 88.76 nm^2^ for the extended state. Thus, the computational results compare quite well with the experimental data regarding the more compact form. This finding is in line with expectations from classical MD simulations, which are likely to capture conformational properties of collapsed forms, leaving, however, extended states quite unexplored. The *in-silico* SAS values identify two groups of different compactness within the collapsed state. One group comprises conformations that mostly derive from the energy basins B, C, D, E, F, with more compact and globular structures, characterized by the lowest SAS values (53–55 nm^2^). The second group includes mainly structures sampled in the energy basins A, G, H, I, L. They display slightly less compact conformations, characterized by slightly higher average SAS values (58–65 nm^2^). These differences might reflect structural heterogeneity within the collapsed state that could not be detected by CSD or ion-mobility analysis (Brocca et al., 2011b). Nevertheless, it cannot be ruled out that the group with lower SAS values could represent an artifact due to a bias for overcompaction of unfolded proteins common to the current force fields (Click et al., 2010; Knott and Best, 2012).

**Figure 8:**
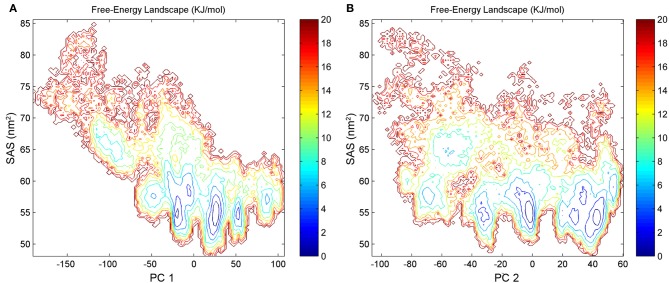
**FEL representations calculated using the first (A) or the second (B) PCs and SAS values as reaction coordinates**. The free energy is given in kJ/mol and indicated by the color code shown on the figure.

To better describe the SAS profiles of the Sic1 KID states in our MD ensemble, the FEL was also calculated using the SAS itself and the first and second PCs as reaction coordinates (Figures 8A,B). These FEL representations also point out the presence of several substates of different compactness. The most populated one is characterized by a SAS value around 55 nm^2^ and corresponds to the compact conformations discussed above (from basins B, C, D, E, F).

**Figure 9:**
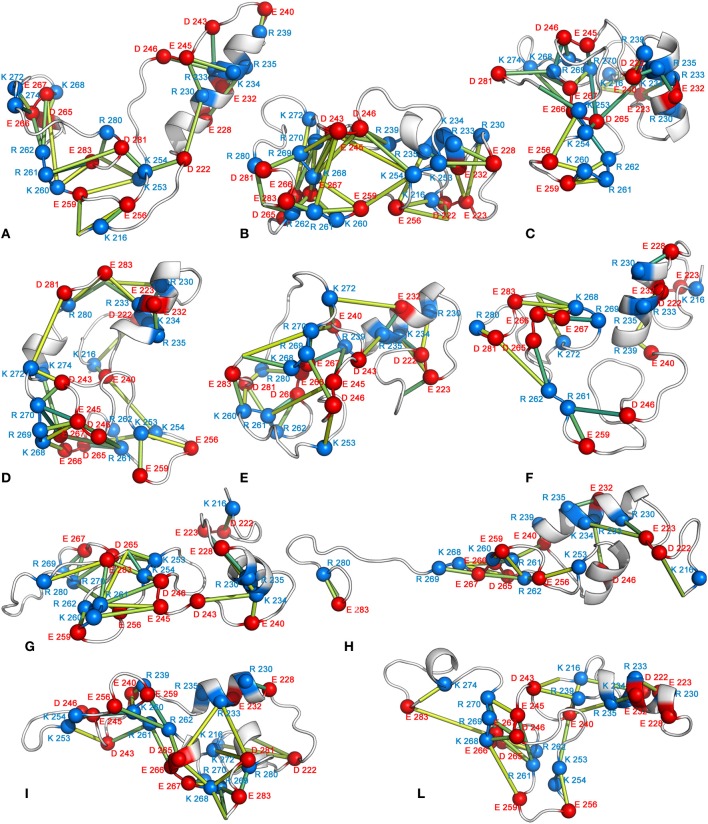
**Networks of salt bridges**. The 3D average structure of each FEL basin is shown as cartoon, and the Cα atoms of the acidic and basic residues involved in salt bridges are shown as red and blue spheres, respectively. The Cα atoms of the interacting residues are connected by sticks of different shades of color depending on the interaction persistence (from yellow to green for increasing persistence values).

To investigate the driving force that likely promotes structural compaction in Sic1 KID, the different types of non-covalent interactions were examined for each FEL basin. In particular, salt-bridge, aromatic, hydrophobic, and amino-aromatic interactions were analyzed for persistence over the simulation time. This analysis identifies salt bridges as the major factor stabilizing compact structures, with a large number of basic and acidic residues involved in multiple interactions (Figure 9). As typical for IDPs, Sic1 KID has a low mean hydrophobicity. In spite of the presence of several charged residues, often consecutive in the amino acid sequence, it also displays quite low net charge per residue (Brocca et al., 2011b). These features are consistent with the propensity displayed by Sic1 KID for globular states (Mao et al., 2010; Brocca et al., 2011a,b).

**Figure 10:**
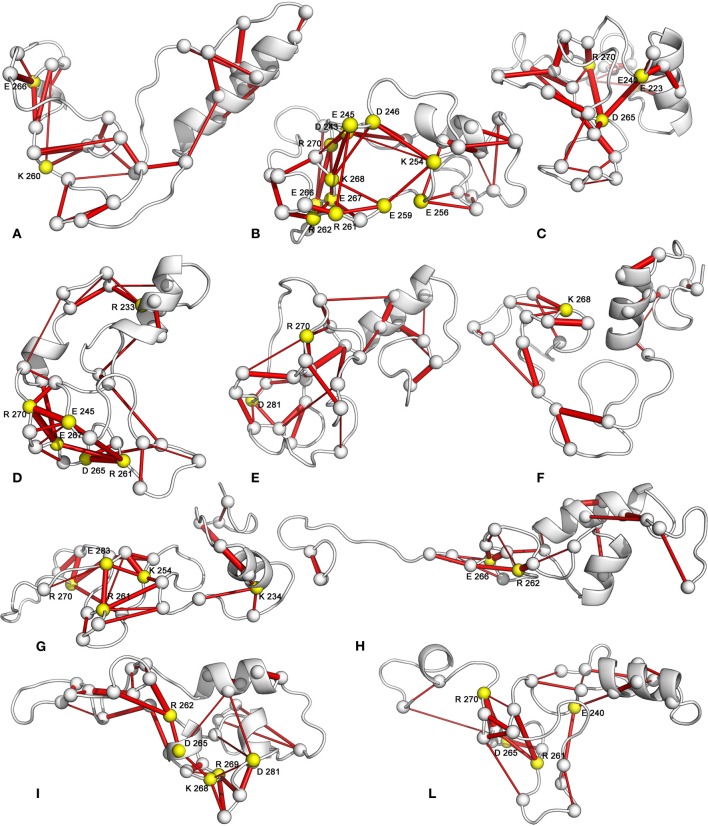
**Hub residues in salt-bridge networks**. The 3D average structure of each FEL basin is shown as cartoon, and the Cα atoms of the residues involved in salt bridges are shown as spheres. The Cα atoms of the interacting residues are connected by sticks, whose thickness is proportional to the persistence of the interaction. Hub residues, defined as those involved in at least three different salt-bridge interactions, are highlighted in yellow.

In Sic1 KID, the salt-bridge networks have a transient nature and each conformational state is mainly stabilized by different pairs of interacting residues. Nevertheless, the analysis of salt-bridge networks in the MD ensemble identifies a subset of residues acting as hubs in the networks (Figure 10). They are likely to represent important residues in the development and maintenance of tertiary structure (Vishveshwara et al., 2009; Angelova et al., 2011). Despite the similar number of pairwise electrostatic interactions, the most compact conformations (basins B, C, D, and E) have salt-bridge networks characterized by more highly interconnected residues than the less compact states (basins A, G, H, and L) (Figures 9, 10). The number of hub residues is generally greater for the more globular conformations (see for example structures from basins D and B) (Figure 10 and Table 3). Interactions involving hub residues in the less compact conformations have lower persistence than those in compact structures (*data not shown*). Some of the hub residues are shared by several compact structures, in particular R270, K268, E267, E245, R261, and D265. The analysis of the major paths connecting charged residues in the graph points out that hubs are also highly interconnected to each other in the compact states, showing multiple paths connecting them with high persistence values (Figure 10).

**Figure 11:**
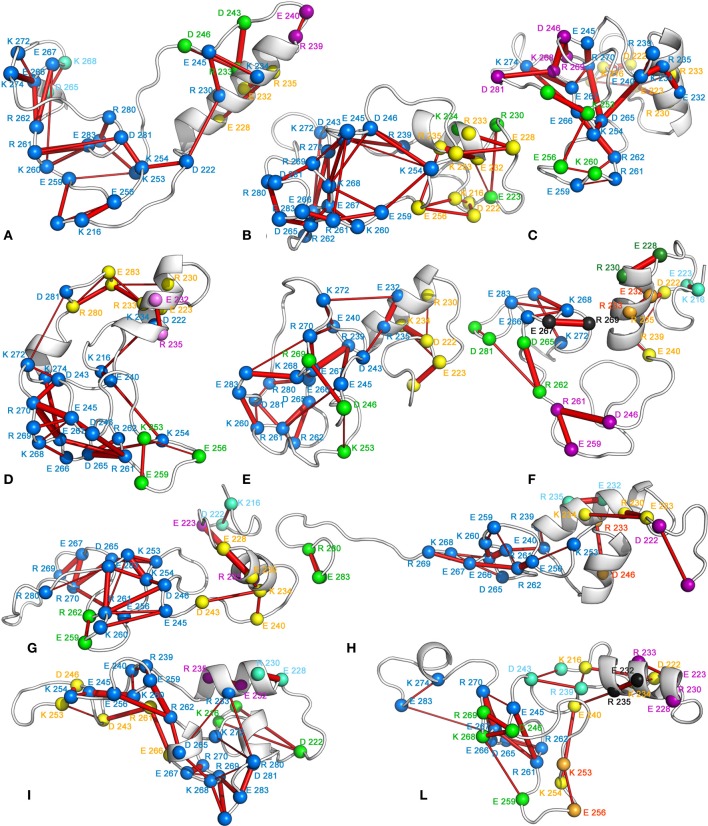
**Sub-networks of salt bridges**. The 3D average structure of each FEL basin is shown as cartoon, and the Cα atoms of the residues involved in salt bridges are shown as spheres. The Cα atoms of the interacting residues are connected by sticks, whose thickness is proportional to the persistence of the interaction. The single sub-networks are represented by a color code according to their size blue-yellow-green-purple-cyan-orange-black-dark green, going from the largest (21 residues) to the smallest one (2 residues).

Sub-networks of salt bridges have also been identified (Figure 11). Less compact states show a greater number of small and poorly connected sub-networks. In fact, these are generally composed by isolated salt bridges or three/four-node networks. On the contrary, the globular states generally feature three major sub-networks, composed by a higher number of well-interconnected residues. These sub-networks include mainly residues from 240 to 280.

**Figure 12:**
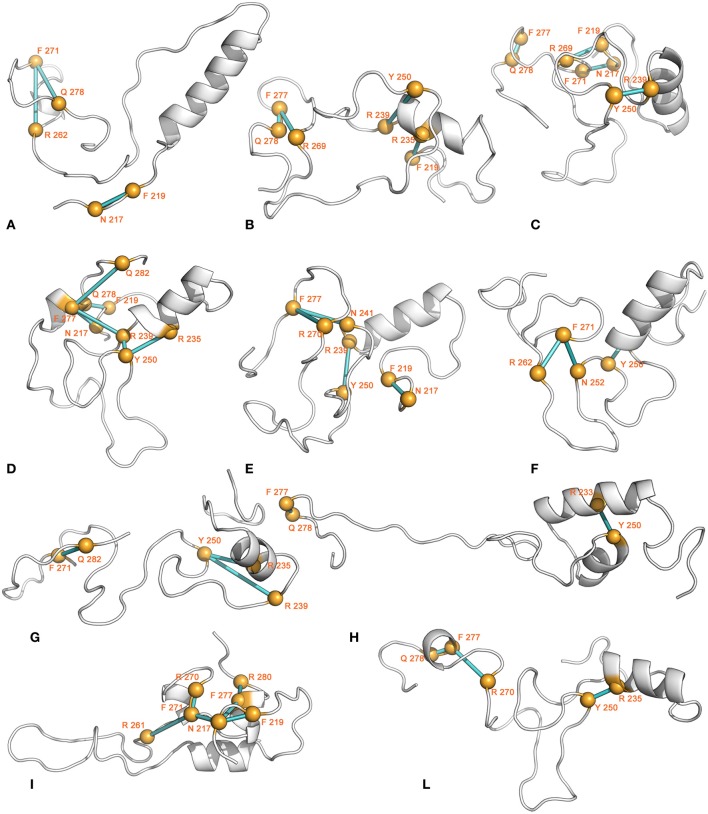
**Amino-aromatic interactions**. The 3D average structure of each conformational basin is shown as cartoon, and the Cα atoms of the residues involved in amino-aromatic interactions are shown as yellow spheres. The Cα atoms of the interacting residues are connected by sticks of different shades of color, depending on the interaction persistence (from cyan to blue for increasing persistence).

Some amino-aromatic interactions could also be detected, even if generally characterized by very low persistence. Among the residues involved in amino-aromatic interactions with highest persistence, we can find Y250, F271, R239, and R235 (Figure 12). Aromatic-aromatic interactions are absent, with exception of basins B and I, where interactions involving F277 and other phenylalanines can be detected (*data not shown*).

Hydrophobic-interaction networks are also present, principally in conformations derived from basins C and I, but they are characterized by very low persistence (*data not shown*). Moreover, an additional search for hydrophobic interactions, lowering the distance cutoffs from 0.5 to 0.45 nm, causes a complete loss of these interactions.

A marginal role of hydrophobic interactions in the stabilization of Sic1 KID compact structures is also indicated by ESI-MS experiments (Figure 7). The spectrum of the protein in 50 mM ammonium acetate, pH 6.5 is characterized by the aforementioned bimodal CSD, indicating coexistence of compact and extended states. The addition of acetonitrile or methanol up to 50% (the highest tested concentration) does not significantly affect the CSD, leaving clearly preserved the compact state. On the contrary, the compact form can be readily *denatured* by acids, as previously shown (Brocca et al., 2011b).

## Discussion

The heterogeneous and dynamic nature of IDPs makes structural characterization of their unbound state highly challenging. Although MD force fields have been developed to simulate protein folding, they have also proven useful to characterize the conformational ensembles of IDPs and unfolded proteins (Espinoza-Fonseca, 2009a; Cino et al., 2011; Arrigoni et al., 2012; Ganguly et al., 2012; Lindorff-Larsen et al., 2012; Knott and Best, 2012), especially when computational results are complemented by biophysical data. These studies contribute to enforce the applicability of classical MD to complex molecular ensembles. Nevertheless, analysis of dynamic and heterogeneous systems such as IDPs has to face limits in force field accuracy and sampling efficacy (Esteban-Martin et al., 2012). Thus, while helping description of globular IDP states, classical MD simulations are not adequate to describe the actual equilibrium between extended and compact conformations. This complementary information can be provided by experimental assessment of species distributions, for instance by MS (Kaltashov and Abzalimov, 2008) or NMR investigation (Esteban-Martin et al., 2012; Schneider et al., 2012).

We employ here atomistic, explicit-solvent MD simulations integrated by experimental data (Brocca et al., 2011b) to provide a first atomic-level description of the conformational ensemble of compact states of the isolated Sic1 KID fragment. The results indicate that, in spite of its strong propensity for structural disorder, Sic1 KID can explore compact conformations, with considerable secondary and tertiary structure. The extents of secondary structure and solvent accessibility derived by the simulations are in good agreement with experimental results obtained by FT-IR spectroscopy and ESI-MS (Brocca et al., 2011b). The conformational ensemble of Sic1 KID reveals a highly dynamic behavior, populating several different conformations. Also local conformations, such as helical IFSUs, are likely to be dynamic.

The present results could also be interpreted in the light of the structural and functional relation to the mammalian p21 and p27 KID domains (Barberis et al., 2005). In fact, it has been shown that p27 can replace Sic1 in yeast cells (Barberis et al., 2005) and that Sic1 KID can functionally interact with mammalian Cyclin A-Cdk2 inhibiting its kinase activity. The interaction between p27 and cyclin A/Cdk2 has been investigated suggesting a two-site, sequential binding process, in which p27 KID first interacts at one end with cyclin A (sub-domain D1) and then binds to Cdk2 by the other end (sub-domain D2), wrapping the central helical region (sub-domain LH) around the cyclin/kinase complex (Sivakolundu et al., 2005; Galea et al., 2008; Espinoza-Fonseca, 2009b; Otieno et al., 2011). The present results point to the stretch between E223 and L238 as the most persistent α-helix of Sic1 KID, while a shorter and transiently populated α-helix approximately maps between residues I244 and I248. Although it is difficult to identify the exact boundaries of the helical regions by MD simulations of such a highly heterogeneous system, these α-helical regions of Sic1 correspond to the p27 LH sub-domain (residues 38–60), according to the structural alignment of the two KID domains (Barberis et al., 2005). This sub-domain has been identified as an IFSU also in p27 (Sivakolundu et al., 2005) and it is thought to play a role tethering the D1 to the D2 sub-domain and enhancing the overall ΔG of binding (Otieno et al., 2011). The corresponding and structurally similar region of Sic1 KID might, therefore, promote binding to the cyclin/kinase complex by a similar mechanism. More studies will be needed to test this hypothesis, and further biochemical investigation will be needed to characterize the physiological intermediates of Sic1 binding.

Moreover, according to the MD scenario reported here, electrostatic interactions seem to be the major determinant of structural compaction in the isolated Sic1 KID. This result of the MD simulations is supported by experimental evidence by ESI-MS. Furthermore, this conclusion is consistent with the low mean hydropathy and low mean net charge per residue of this protein (Brocca et al., 2009, 2011b) and is in agreement with the current view on the importance of charged residues defining IDP structural and functional properties (Uversky et al., 2000).

Electrostatic interactions could also be relevant *in vivo*, in relation to the multiple phosphorylation events that regulate Sic1 interactions (Nash et al., 2001; Mittag et al., 2008; Koivomagi et al., 2011). By altering short- and long-range electrostatic interactions, phosphorylation could effectively modulate the conformational properties of this IDP, even far from the site of the modification (Johnson and Lewis, 2001; Arrigoni et al., 2012).

In particular, the present analysis points out that the globular states of Sic1 KID are stabilized by interconnected networks of electrostatic interactions with a few hub residues common to different conformations and involved in multiple paths. R270, K268, E267, E245, R261, and D265 emerge as the most relevant ones. These residues represent good targets for mutagenesis experiments to further explore the role of such networks in Sic1 KID structure. Although our results do not hint to a major role of hydrophobic residues in intramolecular networks, these could still contribute to global compaction of the domain. Further experiments will be necessary to investigate their structural role.

In conclusion, the here provided experimental and computational evidence indicates that Sic1 KID, though highly disordered, can acquire transient secondary and tertiary structure populating compact conformations.

### Conflict of interest statement

The authors declare that the research was conducted in the absence of any commercial or financial relationships that could be construed as a potential conflict of interest.

## Research Topic:

From structural to molecular systems biology: experimental and computational approaches to unravel mechanisms of kinase activity regulation in cancer and neurodegeneration

## Topic Editor:

Matteo Barberis, Humboldt University Berlin, Germany; Max Planck Institute for Molecular Genetics, Berlin, Germany
